# Clinical efficacy analysis of laparoscopic uterine artery pre-ligation combined with hysteroscopic curettage in the treatment of type II cesarean scar pregnancy

**DOI:** 10.3389/fmed.2023.1234499

**Published:** 2023-08-03

**Authors:** Dan Teng, Han Gao, Yanli Li, Tingzhu Meng, Xiuting Shi, Jie Shi

**Affiliations:** ^1^Medical College, Wuhan University of Science and Technology, Wuhan, China; ^2^Department of Gynecology Hubei Province Maternal and Infant Health Hospital, Tongji Medical College, Huazhong University of Science and Technology. Wuhan, China

**Keywords:** cesarean scar pregnancy, uterine artery pre-ligation, uterine artery embolism, intrauterine adhesions, pregnancy outcome

## Abstract

**Objective:**

To explore and evaluate the clinical therapeutic effect of laparoscopic uterine artery pre-ligation combined with hysteroscopic curettage in the treatment of type II cesarean scar pregnancy.

**Methods:**

This study analyzed the clinical data of patients with cesarean scar pregnancy (CSP) in the Maternal and Child Health Hospital of Hubei Province from 2018 to 2022. A total of 134 patients with type II cesarean section were enrolled, out of which 78 patients were included in the final analysis. Treatment included either uterine artery embolization (UAE) combined with hysteroscopic curettage (*n* = 37 patients) or laparoscopic uterine artery pre-ligation (LUAP) combined with hysteroscopic curettage (*n* = 41 patients). The demographic and clinical characteristics of these two groups were recorded, and their short- and long-term complications on follow-up were compared. For patients with subsequent fertility requirements, we followed up these patients for 2 years after surgery, then collected and analyzed the compared subsequent pregnancy outcome.

**Results:**

We found no significant discrepancies in the success rate of operation, length of hospital stay, and intraoperative blood loss between the two different operation modes. The cost of LUAP was significantly lower than that of UAE. Furthermore, the incidence of short-term postoperative complications such as fever and pelvic pain was lower in patients treated with LUAP than in those treated with UAE. In terms of long-term postoperative complications, the recovery time for menstruation in the LUAP group (49.81 ± 11.47) was earlier than that in the UAE group (34.90 ± 7.41) (*p* < 0.05). Additionally, 4.9% of patients in the LUAP group had decreased menstrual flow, while 59% of patients in the UAE group had a marked decrease in menstrual flow, and the incidence and severity of intrauterine adhesions were significantly lower in the LUAP group than in the UAE group(*p* < 0.05). Consistent with the aforementioned observations, patients treated with LUAP had better postoperative re-pregnancy outcomes than those treated with UAE.

**Conclusions:**

Based on the findings, LUAP combined with hysteroscopic curettage is a safe and effective surgical scheme for the treatment of type II CSPs. In addition, compared with UAE, LUAP is associated with a lower surgical cost, fewer short and long-term complications, and better postoperative pregnancy outcomes. Thus, it should be widely applied in patients with type II CSPs.

## 1. Introduction

Cesarean scar pregnancy (CSP) is defined as a gestational embed either on the scar created by a previous cesarean delivery or within the anterior wall myometrial defect or niche (1). In recent years, the incidence of CSP has continued to increase, representing approximately 1.15% of all pregnancies (2). It is associated with serious complications including placenta accreta, pensive placenta previa, uterine rupture, and even maternal death (3). Transvaginal ultrasound has been found to be the most practical and effective method for diagnosing CSP (4). In the “Chinese expert consensus on cesarean scar pregnancy,” according to the direction of growth of the gestational sac (GS) and the thickness of the myometer between the GS and the bladder, CSPs have been split into three types. Nonetheless, the pathogenesis of CSP remains unclear, and the optimal management remains uncertain. According to the guidelines of the Society for Maternal-Fetal Medicine, early detection, early treatment, and prompt individualized treatment are recommended, while avoiding expectant therapy and simple curettage. There are various treatment methods for CSP in clinics. These methods can be divided into medically conservative treatments and surgical treatments. Conservative treatment has several disadvantages, including long duration, persistent risk of uterine rupture and bleeding, and the need for additional treatments. Patients undergoing transvaginal lesion resection are mostly treated with methotrexate before the operation, which has the risk of gastrointestinal reactions, liver function damage, bone marrow suppression, and other adverse reactions. Furthermore, the surgical procedure is difficult, requires a highly skilled surgeon, and has few clinical applications. In addition, high-intensity focused ultrasound and double-balloon compression of the uterine cavity have been mentioned in the literature, but these procedures are rarely used, and their surgical effect and safety are difficult to evaluate. Laparoscopic lesionectomy is the preferred surgical method; its safety and efficacy have been confirmed, with a success rate of up to 85%, especially for patients with pregnancy needs. With this method, cesarean section scar repair can be performed (5). All of the above-mentioned surgical procedures have potential risks such as bleeding, uterine rupture, uterine arteriovenous fistula, and even hysterectomy (6). However, regardless of the method applied, multiple treatment measures for CSP may cause massive intraoperative blood loss because of the highly vascular nature of the site of pregnancy (7). Consequently, uterine artery embolization (UAE) is an option used in clinical practice to prevent intraoperative blood loss. However, UAE carries a risk of post-embolization syndrome, including complications such as fever, ovarian function damage, irregular vaginal bleeding, lower abdominal pain, and intrauterine adhesions (8). To reduce the incidence of post-embolization syndrome, we adopted the laparoscopic uterine artery pre-ligation (LUAP) procedure to temporarily block the blood supply to the uterus. This procedure is less invasive than UAE. It uses only sutures and does not require the use of special equipment. In addition, LUAP blocks the blood flow for only a few minutes, thereby potentially reducing the incidence of postoperative complications due to interrupted blood supply. It also ensures the reduction of massive intraoperative bleeding. Hence, we used the LUAP approach for the first time in the treatment of type II cesarean scar pregnancy and evaluated its safety and efficacy.

## 2. Materials and methods

### 2.1. Patient selection and data collection

**Figure 1:**
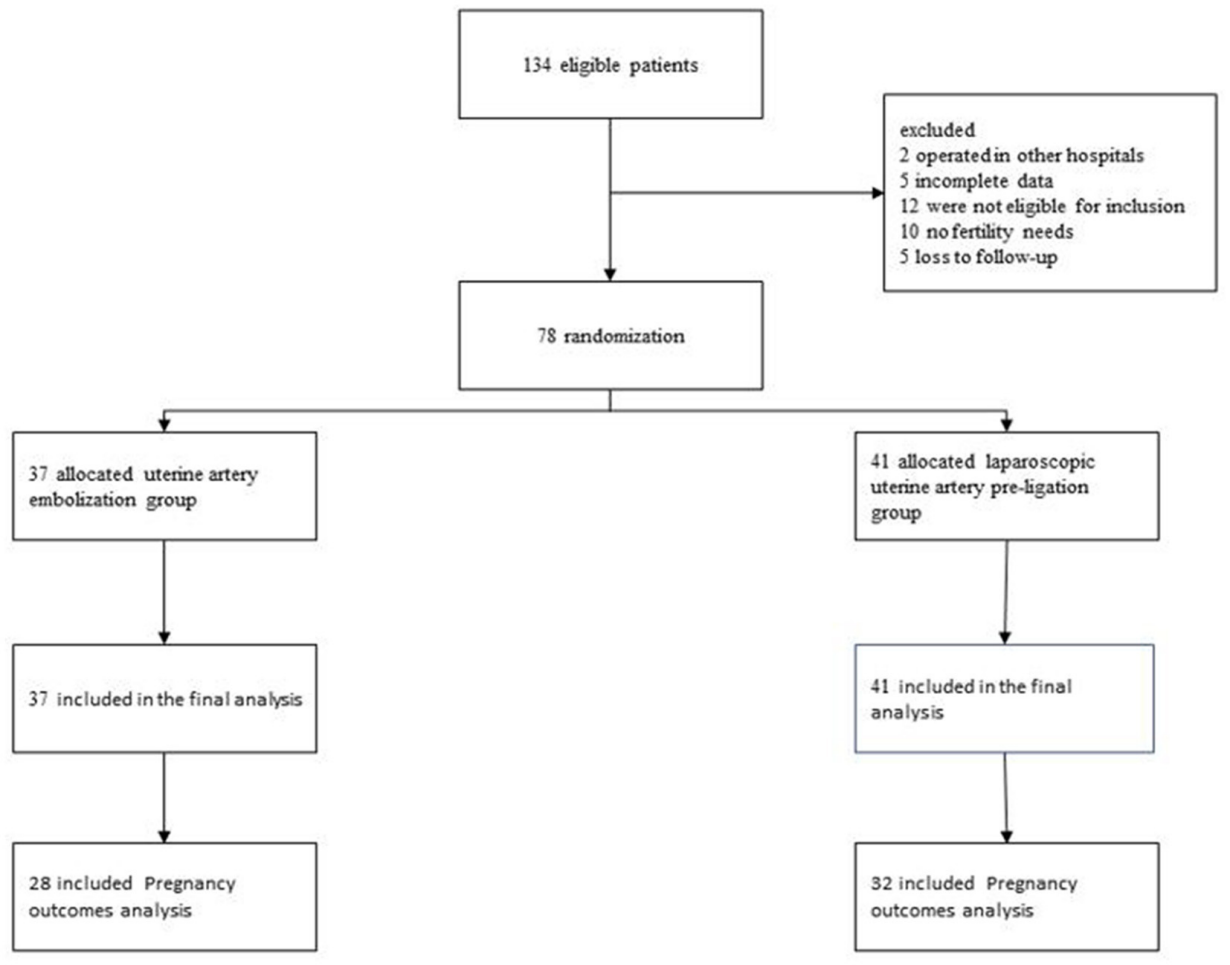
Flow diagram of study.

The ethics committee of Hubei Maternal and Child Health Hospital sanctioned the retrospective study. All patients ruled out contraindications before the operation; they were fully informed of the condition and the possible risks of the operation and signed the operation notification form. Data from 134 patients with type II CSP treated in the hospital during 2018–2022 were obtained and collected from the hospital's record room. The collected information comprised demographic data, medical history, prior experience of cesarean section, abortion history, and intraoperative indicators. The patients were followed up via regular telephone contact for 24 months; short- and long-term complications and postoperative pregnancy outcomes were documented following their discharge. The flow diagram of the study is shown in Figure 1.

### 2.2. Surgery method

**Figure 2:**
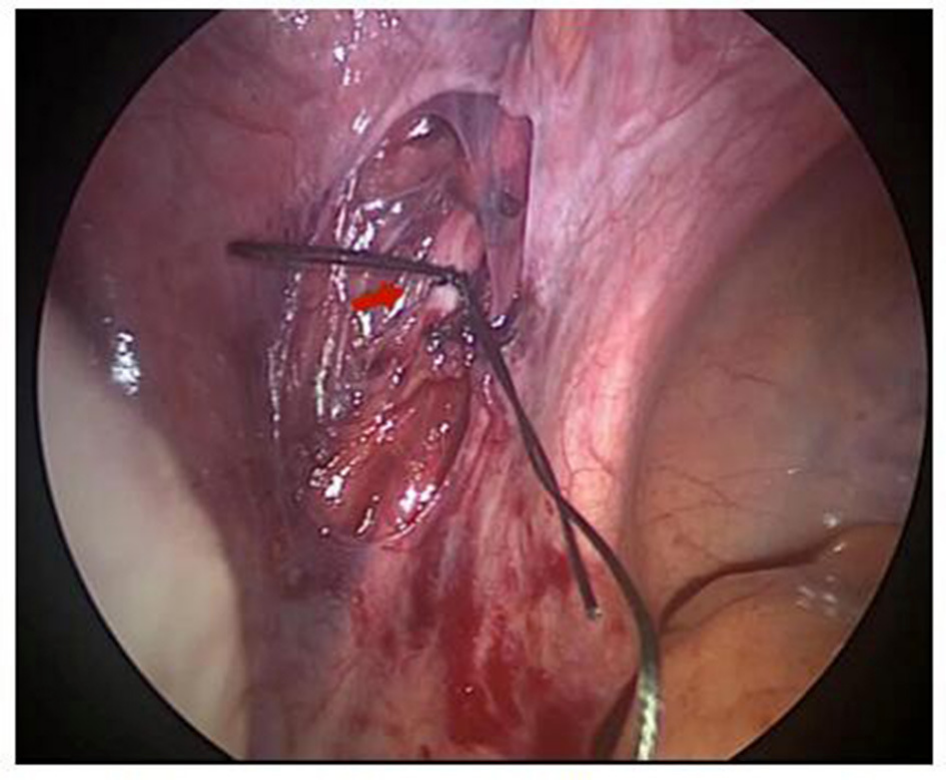
The uterine artery was preligated laparoscopically and the red arrow indicates the uterine artery.

In the LUAP group, the retroperitoneum was opened, and bilateral ureters were identified. Approximately 1.5 cm of the initial end of the uterine artery was free, and bilateral uterine arteries were relegated with a slipknot with 1-0 absorbable thread (Figure 2). Then, the uterine arteries on both sides were blocked temporarily. The peritoneum was separated at the lower part of the uterus where the bladder is inverted, and a slight bulge was observed in the scar of the lower uterine segment. Subsequently, curettage was performed transvaginally under laparoscopic monitoring. The uterine cavity and uterine incision diverticulum were examined by hysteroscopy. The 1-0 silk thread was taken out under a laparoscope, and the color of the uterus was observed. The time from uterine artery ligation to blood flow recovery was approximately 10 min. All the tissues were scraped out of the uterine cavity after the operation and sent to the pathology department to exclude hydatidiform moles.

In the LUAP group, the right transfemoral approach was selected for artery access. The uterine artery was selectively catheterized with a 5F-Y ashier catheter and embolized with gelatin sponge particles of sizes 560–700 um. Angiograms were conducted to confirm whether the occlusion of blood flow was complete. Hysteroscopic curettage was performed 24 h after UAE to remove pregnant tissues. All the tissues were scraped out of the uterine cavity after the operation and sent to the pathology department to exclude hydatidiform moles.

### 2.3. Patient follow-up

We collected the basic information of patients after surgery (including age, clinical symptoms, the number of induced abortions, days of menorrhea, the number of cesarean sections, the diameter of the gestational sac, and surgical cost). We then followed up on the short-term postoperative complications, including the evaluation of procedural success rate, monitoring intraoperative bleeding, and measuring hospital stay duration. We documented the short-term complications after surgery, such as fever and pain. We conducted outpatient follow-up to calculate the recovery time of the first menstruation, menstrual volume alteration, and the duration of pain in the pelvic area. Two months later, the patients were reexamined by hysteroscopy, and the status of intrauterine adhesion was evaluated [the score of intrauterine adhesion was based on the American Reproductive Society (AFS) score in 1988]. Finally, patients who had pregnancy needs were followed up for 24 months to observe their pregnancy outcomes (including delivery conditions, preterm birth, abortion, re-scarred pregnancy, and placental abnormalities).

### 2.4. Statistical analysis

The mean standard deviation (±s) is used to express the measurement data. Basic information and clinical characteristics of patients were tested by performing a two-sample *t*-test with normal distribution. Non-parametric tests were performed when the data were skewed and non-normally distributed. The *X*^2^ test was performed to compare the count data, and Fisher's exact test was performed when *n* < 5. Statistical analysis was performed using SPSS 26.0. Statistical tests performed were two-sided, and *p* < 0.05 was considered significantly different.

## 3. Results

### 3.1. Basic information and characteristics of patients

The study included 134 patients with type II CSP, out of which 78 patients were included in the final analysis. Among them, 31 patients experienced minimal vaginal bleeding before the operation, two patients had vaginal bleeding and abdominal pain before the operation, and the remaining had no clinical symptoms and were diagnosed by transvaginal ultrasound. Table 1 displays the baseline clinical characteristics of CSP. Age, clinical symptoms, number of induced abortions, days of menorrhea, number of cesarean sections, or the diameter of the gestational sac did not significantly differ between the two groups. However, the operational expenses of LUAP were considerably lower than those of UAE, and this difference was statistically significant.

**Table 1:** Comparison of the baseline characteristics of CSP patients.

**Groups**	**UAV (*n =* 37)**	**LUAP (*n =* 41)**	***X* ^2^**	***p*- value**
Age (y)	32.73 ± 3.78	32.46 ± 4.84	0.278	0.781
Clinical symptoms	45.9% (17/37)	39% (16/41)	0.382	0.647
Number of induced abortions	1.68 ± 1.13	1.49 ± 1.26	/	0.317
Number of cesarean sections	1.41 ± 0.55	1.35 ± 0.54	/	0.443
Days of menopause (d)	51.90 ± 15.78	45.54 ± 9.92	1.355	0.179
Diameter of gestational sac (cm)	2.85 ± 1.13	3.27 ± 1.33	1.498	0.138
surgical cost ($)	16,213.49 ± 2,502.94	19,388.49 ± 8,706.60	2.140	0.038

### 3.2. Clinical features and short-term complications

No patients were missed during the follow-up evaluation. The operation of patients in both groups was smooth without serious complications such as a massive hemorrhage or ureteral injury. The surgical success rate of patients in both groups was 100%. The two groups did not show any notable distinction in terms of the length of hospital stay, the amount of blood loss during surgery, and the uterine perforation rate. Out of the 37 patients who underwent UAE, three patients experienced mild fever following the procedure. Furthermore, 25 patients experienced moderate to severe pain after their surgery. In the long-term follow-up, 17 patients had pelvic pain for more than 2 weeks, and 6 patients had pelvic pain for 6 months without obvious relief. In the LUAP group, only one patient experienced low fever, and none experienced long-term pelvic pain (Table 2). In addition, in the LUAP group, postoperative pain disappeared within 1 day without the requirement of any intervention. However, the duration of pain was longer in the UAE group, with most patients requiring nonsteroidal anti-inflammatory drugs and even opioids for pain relief, than in the LUAP group. In conclusion, the incidence and severity of short-term complications were lower in the LUAP group than in the UAE group.

**Table 2:** Clinical features and short-term complications.

**Variables**		**UAV (*n =* 37)**	**LUAP (*n =* 41)**	***X* ^2^**	***p*-value**
Success rate (%)		100% (37/37)	100% (41/41)	0.278	1.000
Duration of hospital stay (d)		7.29 ± 0.93	7.22 ± 0.89	0.371	0.712
Intraoperative blood loss (ml)		22.29 ± 5.72	21.95 ± 9.21	1.97	0.854
Perforation of the uterus (*n*)		8.1% (3/37)	2.4%(1/41)	/	0.353
Complication rate (%)		75.6% (28/37)	4.8%(2/41)	41.187	0.000
Fever (*n*)		3	1	/	0.034
Postoperative pain (*n*)		25	1	37.123	0.000
Postoperative pain score	< 4	2	41	37.123	0.000
	5–6	13	0		
	>7	10	0		

Postoperative pain: using a numerical rating scale (NRS), 4 or less was defined as mild pain (pain did not affect sleep), 4–6 was defined as moderate pain, and 7 or more was defined as severe pain (could not sleep due to pain or woke up in pain).

### 3.3. Long-term postoperative complications

The comparison of long-term postoperative complications between the two groups is presented in Table 3. The first appearance of menstruation after the operation occurred earlier for patients in the LUAP group than for patients in the UAE group. Furthermore, the two groups differed significantly in terms of changes in menstrual volume, duration of pelvic pain, and intrauterine adhesion score. In the LUAP group, the menstrual volume of two patients decreased by 1/3 compared with the preoperative volume, and one patient experienced postoperative pelvic pain that receded within 1 day. Throughout the 6-month monitoring period, mild intrauterine adhesions were observed only in two patients. Contrarily, the menstrual volume of 22 patients in the UAE group decreased by 1/3 compared with the preoperative volume. More importantly, 23 patients experienced chronic pelvic pain, six of whom had persistent chronic pelvic pain without relief. Furthermore, 21 patients had moderate to severe intrauterine adhesions, of whom five patients had to undergo surgical treatment for severe intrauterine adhesions. Thus, compared with UAE, LUAP demonstrated significant advantages in restoring menstruation and reducing the incidence of postoperative pelvic pain and intrauterine adhesions.

**Table 3:** Long-term postoperative complications.

**Variables**		**UAV (*n =* 37)**	**LUAP (*n =* 41)**	***X* ^2^**	***p*-value**
Time of first menstrual resumption		34.90 ± 7.41	49.81 ± 11.47	6.737	< 0.001
Changes in menstrual volume	Decrease 1/3 Decrease 1/2	22 (22/37) 10 (10/37)	2 (2/41) 0 (0/41)	52.677	0.000
Duration of pelvic pain	< 1 Day	2 (2/37)	1 (1/41)	31.123	0.000
	>2 week	17 (17/37)	0 (0/41)		
	>6 months	6 (6/37)	0 (0/41)		
Intrauterine adhesions score	1~4	2 (2/32)	2 (2/41)	29.304	0.000
	5~8	12 (12/32)	0 (0/41)		
	9~12	9 (9/32)	0 (0/41)		

Uterine adhesions were scored with reference to the 1988 American Fertility Society (AFS) score: 1–4 for mild adhesions, 5–8 for moderate adhesions, and 9–12 for severe adhesions.

### 3.4. Postoperative pregnancy outcome

We followed up two groups of patients with pregnancy intentions for up to 24 months, as presented in Table 4. We found that the pregnancy rate was comparable in both groups, without any notable differences in the outcomes. In the UAE group, 37 patients had pregnancy intentions, 28 of whom had successful pregnancies. In the LUAP group, 37 patients had pregnancy intentions, 32 of whom had successful pregnancies. The rate of CSP patients who became pregnant again after undergoing UAE treatment was 75.6%, and that of CSP patients who underwent LUAE treatment was 78%. However, the rate of CSP in the UAE group was 4.571 times that in the LUAP group. In the LUAP group, there were 32 cases of successful pregnancies, including 23 full-term birth cases (cesarean section), two preterm birth cases, two pregnancy state cases, and two spontaneous abortion cases. All 28 cases in the UAV group were CSPs (6 cases were placental abnormalities), 11 cases were full-term deliveries, four cases were premature deliveries, and five cases were spontaneous abortions.

**Table 4:** Surgical pregnancy outcomes.

**Postoperative pregnancy outcome**	**UAV (*n =* 37)**	**LUAP (*n =* 41)**	***X* ^2^**	***p*-value**
Number of pregnancies	28 (28/37)	32 (32/41)	0.062	0.804
Full-term delivery	11 (11/28)	23 (23/32)	6.459	0.018
Premature birth	4 (4/28)	2 (2/32)	/	0.396^a^
Mid-pregnancy	0 (0/28)	2 (0/32)	/	0.494^a^
Spontaneous abortion	5 (5/28)	3 (3/32)	/	0.454^a^
Re-scarred pregnancy	8 (8/28)	2 (2/32)	/	0.036^a^
Placental abnormalities	6 (6/28)	0 (0/32)	/	0.008^a^

^a^Fisher's precision probability test.

The results revealed that the two groups had no significant differences in the rate of preterm birth, mid-pregnancy, and spontaneous abortion. In addition, six cases of placental abnormalities occurred in the UAE group, and the difference was statistically significant when compared with the LUAP group. Of the six cases, there were two cases of placenta accrete, and four cases of placenta previa. All the cases had different degrees of postpartum hemorrhage, and two patients underwent blood transfusion. Furthermore, two out of four preterm pregnancies were terminated early at approximately 35 and 31 weeks of gestation, respectively, due to placental abnormalities. There was no uterine rupture in the two groups. Based on the above results, the pregnancy outcome of patients in the LUAP group was better than that in the UAE group.

## 4. Discussion

First reported in 1978 by Larsen and Solomon (9), CSP is a specific type of ectopic pregnancy. In the last decade, the detection rate of CSP has gradually increased with the increase in the number of cesarean sections and improvement in the level of ultrasound diagnosis (10). However, a full understanding of the pathogenesis of CSP is lacking. Some studies believe defects in the scar tissue following a cesarean section to be the direct cause. Owing to poor healing of the muscular layer and endometrium at the uterine incision, a sinus or fissure is formed, and in severe cases, even a diverticulum of the uterine incision is formed (11). Some studies reported that the occurrence of CSP is also related to the decrease of local blood supply in the uterine incision, trophoblast invasion of the hypoxic environment, and the chemotactic effect of inflammatory factors. All of these factors work together to induce the implantation of fertilized eggs and implantation in the cesarean section scar (12).

The symptoms and indications of CSP lack specificity. Darwish et al. reported that 47.6% of the patients in their study exhibited no symptoms, 33.3% of the patients experienced vaginal bleeding, and 19.1% experienced abdominal pain along with vaginal bleeding (13). In our study, of the 78 type II CSP patients, 39.7% reported experiencing minor vaginal bleeding, and 10.2% reported experiencing lower abdominal pain along with vaginal bleeding. The remaining patients exhibited no symptoms and were diagnosed by ultrasound examination. If the first pregnancy ultrasound is performed later, the diagnosis may be delayed due to asymptomatic CSP (14). Once the diagnosis is delayed, a CSP can easily cause placenta implantation, which may result in massive hemorrhage and even hemorrhagic shock. Therefore, optimal prognosis can only be achieved through early diagnosis and treatment (15).

To the best of our knowledge, no standardized treatment for CSP has been established. Available therapies include medical interventions, surgery, or a combination of the two. In recent years, experience with the management of CSP has increased, and more patients with CSP are treated by minimally invasive surgery (16). Nonetheless, the optimal surgical options, their efficacy, and the correlated risk factors have yet to be conclusively determined. According to some previous studies, the treatment of CSP with UAE before curettage could help reduce the incidence of massive intraoperative bleeding (17). However, UAE may potentially lead to ovarian function and urinary system damage, causing intrauterine adhesions and even resulting in sepsis and embolic syndrome (18). Considering the above risks, obstetricians and gynecologists have been attempting to find new treatments for CSP. In recent years, laparoscopic resection of lesions has been applied to the treatment of CSP, and its safety and effectiveness have been confirmed by several studies (19, 20). However, direct resection of the lesion under laparoscopy increases the risk of massive intraoperative bleeding. On this basis, in our operation, we added the method of uterine artery pre-ligation to avoid massive intraoperative and postoperative bleeding.

In this study, the efficacy, safety, postoperative complications, and pregnancy outcomes of LUAP in the treatment of type II CSP were evaluated. We compared it with UAE because UAE has been routinely used in the treatment of CSP to prevent massive intraoperative bleeding. We found that both approaches exhibited similar surgical success rates, lengths of hospital stays, rates of postoperative pregnancy, and intraoperative blood losses. The rate of short-term complications in the UAE group was higher than that in the LUAP group (75.6 % vs. 4.8%). Moreover, the total cost of treatment in the UAE group was remarkably higher than that in the LUAP group. After 24 months of follow-up, the recovery time for menstruation in the LUAP group was earlier than that in the LUAP group, and there was no visible reduction in menstrual volume after surgery. In addition, compared with patients in the UAE group, patients in the LUAP group experienced no long-term chronic pelvic pain, and the incidences of intrauterine adhesions were fewer and less severe. Furthermore, pregnancy outcome after surgery was superior in the LUAP group compared with the UAE group.

In the treatment of LUAP, we performed pre-ligation of the uterine artery before hysteroscopic curettage, which can result in a marked decrease in blood loss during surgery in patients. The removal of scar tissues under direct laparoscopic vision can ensure the complete excision of CSP lesions, significantly reducing the risk of scar pregnancy and placental abnormalities in subsequent pregnancies. Pre-ligation of the uterine artery for only a few minutes of blood flow occlusion can minimize the negative impact on the direction of the uterine and ovarian vessels and drastically reduce the patient's postoperative complications. Compared with UAE, LUAP treatment is a minimally invasive and comfortable treatment modality without the risk of lower limb immobilization.

In this study, there was no significant difference in intraoperative blood loss between the two groups. In the UAE group, three patients experienced mild fever following the procedure. We hypothesized that this fever could be attributed to an inflammatory reaction *in vivo* triggered by the embolization material, specifically the gelatin sponge used during the procedure. Although intraoperative blood flow occlusion with uterine artery pre-litigation was limited to a few minutes, minimizing the impact on surrounding tissues and reducing the incidence of postoperative complications in patients are necessary. Compared with UAE, LUAP was advantageous in diminishing postoperative complications.

Ovarian insufficiency, intrauterine adhesions, and amenorrhea are potential late complications of UAE (21). In our study, the UAE group had a longer recovery time for menstruation than the LUAP group and a marked reduction in menstrual volume. In the UAE group, 23 patients experienced intrauterine adhesions of varying degrees, and five patients underwent hysteroscopic surgery due to severe intrauterine adhesions. In contrast, only two patients in the LUAP group experienced mild intrauterine adhesion, and most patients had no intrauterine adhesions. Although patients had a history of curettage, which could also cause intrauterine adhesions, no intrauterine adhesions were found in either group preoperatively, and there was no difference in the number of induced abortions between the two groups. Thus, the potential long-term negative impacts of LUAP were considerably low compared with UAE.

We paid attention to not only the occurrence of severe complications but also the preservation of patients' fertility. A study involving 398 pregnancies following UAE reported the following risks: malpresentation (17%), cesarean delivery (58%), preterm delivery (28%), small for gestational age (7%), and postpartum hemorrhage (13%) (21). A retrospective analysis of pregnancy outcomes after uterine fibroid embolization suggested a higher risk of miscarriage and a significant increase in postpartum hemorrhage in post-UAE pregnancies (22). In our study, we removed the scar tissue under direct laparoscopic vision after pre-ligation of the uterine artery, which could ensure complete resection of the CSP lesion. Uterine scar repair is performed when necessary, which is important for patients with fertility requirements. We found that both LUAP and UAE can achieve a satisfactory natural pregnancy rate, but the rate of cesarean scar is higher in re-pregnancy after UAE, which increases the risk of scar pregnancy and placental abnormalities such as placenta previa and accreta. Placental abnormalities increase the incidence of preterm birth, and preterm infants often have a worse prognosis than full-term infants. Abnormal placentas are prone to complications with postpartum hemorrhage, which increases the risk of hysterectomy and severely affects women's physical and mental health.

The strength of this study is that it is a comprehensive retrospective cohort study in which we statistically analyzed the clinical efficacy, safety, and pregnancy outcomes of LUAP in patients with type II CSP. We have extensive experience in the treatment of CSP with LUAP combined with hysteroscopic curettage. However, our study also has some limitations. In the follow-up of postoperative complications, we used telephone follow-up, and the conclusions drawn were subjective to some extent. The sample size was small, and some patients are still in long-term follow-up. In addition, patients' ovarian function was not evaluated, and we will conduct prospective studies in the future.

Thus, LUAP was associated with lower surgical costs and lesser equipment requirements than UAE. Above all, it was associated with faster postoperative patient recovery, fewer postoperative complications, and better pregnancy outcomes than UAE for the treatment of type II CSP. Based on this, LUAP should be popularized and used in type II CSP. This surgical approach can, perhaps, be used as an alternative to UAE.

## Data availability statement

The original contributions presented in the study are included in the article/supplementary material, further inquiries can be directed to the corresponding author.

## Ethics statement

Written informed consent was obtained from the individual(s) for the publication of any potentially identifiable images or data included in this article.

## Author contributions

DT performed data management, project management, and writing of the original draft. HG explored the surgical protocol for the study. YL participated in the literature design and validated the analytical methods. TM and XS contributed to the clinical data collection. JS provided the documentation design of the study and revised the manuscript. All authors discussed the results and contributed to the final manuscript.
